# Pre-impact fall detection

**DOI:** 10.1186/s12938-016-0194-x

**Published:** 2016-06-01

**Authors:** Xinyao Hu, Xingda Qu

**Affiliations:** Institute of Human Factors and Ergonomics, College of Mechatronics and Control Engineering, Shenzhen University, 3688 Nanhai Avenue, Shenzhen City, Guangdong Province China

**Keywords:** Fall accidents, Fall prevention, Pre-impact fall detection

## Abstract

Pre-impact fall detection has been proposed to be an effective fall prevention strategy. In particular, it can help activate on-demand fall injury prevention systems (e.g. inflatable hip protectors) prior to fall impacts, and thus directly prevent the fall-related physical injuries. This paper gave a systematical review on pre-impact fall detection, and focused on the following aspects of the existing pre-impact fall detection research: fall detection apparatus, fall detection indicators, fall detection algorithms, and types of falls for fall detection evaluation. In addition, the performance of the existing pre-impact fall detection solutions were also reviewed and reported in terms of their sensitivity, specificity, and detection/lead time. This review also summarized the limitations in the existing pre-impact fall detection research, and proposed future research directions in this field.

## Background

Falls are a major safety concern, especially in older people. Approximately 28–35 % of people aged of 65 and above fall at least once every year [4, 10, 36]. In the US, 70 % of all emergency department visits by people over the age of 75 years were related to falls [39]. The consequences of falls are often devastating. Physical injuries caused by falls, such as hip fracture and head injuries, are often associated with high mortality and morbidity among older people [37]. Besides, older people are likely to develop “fear of repeated falls” after a fall-related incident. This often leads to the loss of mobility and independence [45]. For these and many more reasons, developing an effective fall prevention strategy is imperative to mitigate the harm of falls, especially in older people.

Fall detection, whose main idea is to detect the occurrence of a fall event automatically [16, 33, 46], has been proposed to be an effective fall prevention strategy. Fall detection can be generally classified as post-fall mobility detection and pre-impact detection. Post-fall mobility detection is expected to initiate timely medical assistance for fall victims, and thus avoid unnecessary losses caused by ‘long-lie’ [29, 46]. However, this type of fall detection has an inherent limitation. Falls can be only detected after impacts, so injuries directly caused by fall impacts cannot be prevented.

Unlike post-fall mobility detection, pre-impact fall detection is able to overcome the limitations mentioned above. Pre-impact fall detection refers to the technique that allows falls to be detected before the body hits against the ground (i.e., the body-ground impact). Thus, it can not only help initiate timely medical assistance for fall victims, but also have the potential to help activate on-demand fall protection systems to prevent the physical injuries caused by the body-ground impact. Though on-demand fall protection systems are still in the exploratory phase and not commercially available, the idea of integrating pre-impact fall detection and on-demand fall protection systems have been well accepted as a promising solution to preventing falls in older people [28]. In fact, some researchers have attempted to integrate pre-impact fall detection algorithms with on-demand fall protection systems. For example, Tamura et al. [43] designed a system that integrated the fall detection algorithm with a wearable airbag system which can be inflated to protect fallers from the body-ground impact. Similarly, Shi et al. [41] proposed an inflatable hip proctor that can be triggered by an inertial-sensor-based fall detection system.

Given the above-mentioned reasons for that pre-impact fall detection is superior to post-fall mobility detection, current research has become more focused on developing automatic pre-impact fall detection systems. This paper aimed to give a systematical review on pre-impact fall detection. In this review, we would focus on the following aspects of the existing pre-impact fall detection research: fall detection apparatus, fall detection indicators, fall detection algorithms, and types of falls for fall detection evaluation. In addition, the performance of the existing pre-impact fall detection solutions were also reviewed and reported in terms of their sensitivity, specificity, and detection/lead time.

## Review procedure

The search of articles was based on the following database: Google Scholar, IEEE Xplore, PubMed, Web of Science and Science Direct. To avoid unnecessary omission, the searching criteria was deliberately broadened. We included the keywords of “fall detection”, “fall detector”, “fall event detection”, “fall recognition”, “fall protection”, “fall prevention”, “detect falls”, and “detecting falls”. This initial search yielded an initial collection of 683 papers. After that, screening was carried out to manually identify the studies on pre-impact fall detection only. Further actions were carried out to examine the citations of resulted papers to identify the relevant studies. We excluded papers that gave insufficient information, such as the conference abstract. In addition, one study on pre-impact detection and protection of motorcyclist falls [6] was also excluded as it is irrelevant to falls in older people.

A total of 23 studies on pre-impact fall detection were identified, including 19 journal articles and 4 full papers from conference proceedings. These studies were reviewed in details chronically. The review results were summarized in Table 1.

**Table 1:** Summary on pre-impact fall detection studies

Author	Year	Technology	Fall indicators	Classification method	Fall types	Subjects	Accuracy	Lead time
Wu	[47]	MCS	Horizontal and vertical profile of trunk velocity	Single THD based on the trunk velocity, THD = −1 m/s	3 SMF: tripping, forward fall, backward fall	3 Young adults	Not specified	300–400 ms
Lindemann et al.	[21]	A 3D ACCM, attached to head	Sum-vector of head acceleration	Multiple THD: (1) the sum-vector of 2D (i.e. planar) acceleration from head >2 g; (2) the sum-vector of 3D velocity >0.7 m/s	7 SMF: forward fall, backward fall, sideway fall, fall to the back with hip flexion, fall backwards against a wall, imitation of a collapse, fall while picking up an object	1 Young adult and 1 elderly	SEN = 100 %, SPE not specified	Not specified
Nyan et al.	[33]	Three 3D GYRO, attached to sternum, waist and underarm	Angular rate of sternum, waist and underarm	Multiple THD method: (1) determine the THD (for angular rate) based on a series of ADLs: (2) the THDs were ranged from 100–170 degree/s for different fall indicators	2 SMF: sideway fall; backward fall	10 Young adults	SEN = 100 %, SPE = 92.5–97.5 % (different fall types)	98–220 ms
Bourke et al.	[8]	An integrated 3D ACCM and 3D GYRO, attached to chest	Negative downward vertical velocity of trunk	Single THD based on the trunk velocity, THD = 1.3 m/s	4 SMF: forward fall with legs straight; forward fall with knee flexion; backward fall with knee flexion; side fall to right with knee-flexion	5 Young male adults	SEN = 100 %, SPE = 100 %	150–750 ms; mean = 323 ms
Bourke et al.	[8]	A 3D ACCM, attached to chest	Negative downward vertical velocity of trunk	Single THD based on the trunk velocity, THD = 1.3 m/s	4 SMF: forward fall with legs straight; forward fall with knee flexion; backward fall with knee flexion; side fall to right with knee-flexion	5 Young male adults	SEN = 100 %, SPE = 100 %	150–750 ms, mean = 323 ms
Bourke et al.	[8]	A 2D GYRO, attached to chest	Negative downward vertical velocity of trunk	Single THD based on the trunk velocity, THD = 1.3 m/s	4 SMF: forward fall with legs straight; forward fall with knee flexion; backward fall with knee flexion; side fall to right with knee-flexion	20 Young male adults	SEN = 100 %, SPE = 100 %	Not specified
Nyan et al.	[31]	(1) An integrated 3D ACCM and 3D GYRO, attached to chest; (2) an integrated 3D ACCM and 2D GYRO, attached to right thigh	(1) Sagittal and lateral angles of thigh; (2) correlation of angles between thigh and torso; (3) correlation of angular velocity between thigh and a pre-defined segment	Multiple THD: (1) the THD for thigh angles = ±10 degree; (2) the THD for correlation = 0.99	3 SMF: forward fall; backward fall; side fall	10 Young adults	SEN = 95.2 %, SPE = 100 %	727 ± 190 ms
Nyan et al.	[32]	(1) An integrated 3D ACCM and 3D GYRO, attached to chest; (2) an integrated 3D ACCM and 2D GYRO, attached to right thigh	(1) Torso segment orientation; (2)thigh segment orientation	Multiple THD: (1) 2D thigh orientation THD = ±10 degree; (2) the correlation coefficient between the thigh segment and torso segment orientation >0.8; (3) the correlation coefficient between the body segment orientation and template >0.8	3 SMF: forward fall; backward fall; side fall	13 Young male and 8 young female adults	SEN = 95.2 %, SPE = 100 %	Mean = 700 ms
Wu and Xue	[48]	An integrated 3D ACCM and 3D GYRO, attached to anterior waist	Velocity of inertial sensor frame (i.e. the waist)	Single THD: the THD was first set to −1 m/s and then set to the maximum value of vertical velocity during the non-fall activities	4 SMF: forward fall; backward fall; sideways fall; downward fall	10 Young adults and 8 old adults (ADLs)	SEN = 100 %, SPE = 100 %	70–375 ms
Shi et al.	[41]	An integrated 3D ACCM and 3D GYRO, attached to waist	Waist acceleration and angular velocity	MLM: (1) setting up a database of “falling down” and “non-falling down”; (2) feature selection based on Principle Component Analysis; (3) derive Singular Vector Machine (SVM) classifier by data training; (4) Fall detection by SVM classifier and inflation of airbag for fall detection	2 SMF: lateral fall with fast and slow fall motion	2 Young adults	SEN = 100 %, SPE = 100 %	Not specified
Tamura et al.	[43]	An integrated 3D ACCM and 3D GYRO, attached to waist	Waist acceleration and angular velocity	Multiple THD: (1) waist acceleration <3 m/s^2^ (assumed to be free fall); (2)angular velocity >0.52 rad/s;	3 SMF: forward fall; backward fall; side fall	16 Young adults	SEN = 93.1 %, SPE not specified	Lead time not specified; the detection time = 93–197 ms
Shan and Yuan	[40]	A 3D ACCM, attached to posterior waist	Waist acceleration	MLM: (1) feature selection based on the acceleration data by a discriminant analysis; (2) support vector machine classifier was used	3 SMF: forward fall; backward fall; side fall	5 Young male adults	SEN = 100 %, SPE = 100 %	182–228 ms, mean = 204 ms
Zhao et al.	[49]	A 9-axial IMU (Xsens Tech. B.V.), attached to upper trunk	(1) Acceleration from upper trunk; (2) angular velocity of trunk	Multiple THD: (1) trunk acceleration >7 m/s2; (2) trunk angular velocity >3 degree/s	3 SMF: forward fall; backward fall; side fall	8 Young adults	Not specified	257–329 ms
Liu and Lockhart	[22, 23]	(1) MCS; (2)an IMU attached to trunk; a 3D ACCM attached to thigh	Trunk sagittal angular angle and trunk angular velocity	Individualized THD extracted from a fall discriminant function	Slip induced falls	10 Older adults	SEN = 100 %, SPE = 96.5 %	Mean detection time = 255 ms
Tong et al.	[44]	A 3D accelerometer, attached to chest	Acceleration time series (ATS) from upper trunk	MLM: (1) the ATS extracted from the accelerometer attached to the upper trunk was used to train a Hidden Markov Model (HMM); (2) The output of the HMM, known as the marching degrees (P), was compared with a pre-defined THD, i.e. P1 = 0.334 %; (3) Falls were detected if P < P1	2 SMF: forward fall; side fall	8 Young adults	SEN = 100 %, SPE = 88.75 %	200–400 ms
Martelli et al.	[26]	MCS	Linear acceleration from all body segment	MLM: (1) The linear acceleration of all the body segments was parsed by independent component analysis; (2) a Neural Network was used to classify walking from unexpected perturbations	Slip induced falls	15 Young adults	SEN = 92.7 %, SPE = 98 %	Mean detection, time = 351 ms
Aziz et al.	[1]	A 3D ACCM and a 3-axis GYRO, attached to waist	Waist acceleration, velocity and angular velocity	MLM: (1) use the means and variances of X-, Y- and Z-axis accelerations, velocities and angular velocities to form the 18 features; (2) These features were used in Support Vector Machine to for activities classification	7 Types of falls (involuntary and simulated): slip-induced fall; trip-induced fall; fall from hit or bump by an object or another person; collapse or loss of consciousness; misstep or cross step while walking and; incorrect shift of bodyweight while sitting down on or rising from a chair	10 Young adults	SEN = 93.5−100 %, SPE = 85.6−99.7 %	63–188 ms
Hu and Qu	[13]	MCS	Five fall indicators, tested separately: head vertical acceleration, upper arm vertical velocity, trunk vertical velocity, shank frontal velocity, and head frontal angular velocity	Statistical: (1) an ARIMA model based statistical process control chart was constructed based on historical movement data; (2) The individual-specific control limit based on each fall indicator was used for fall detection	Slip induced falls	60 Young adults	SEN = 88.5−94.7 %, SPE = 92.9−99.2 %	mean detection time = 620–710 ms
Sabatini et al.	[38]	An integrated device with a 3D ACCM, a 3-axis GYRO and a barometric altimeter, attached to right anterior iliac spine	Downward vertical velocity at waist	Single THD based on the trunk velocity, THD = 1.38 m/s	7 Types of falls (involuntary and simulated): slip-induced fall; trip-induced fall; fall from hit or bump by an object or another person; collapse or loss of consciousness; misstep or cross step while walking and; incorrect shift of bodyweight while sitting down on or rising from a chair	25 Young adults	SEN = 80 %, SPE = 100 %	40–300 ms mean = 157 ms
Hu and Qu	[12]	MCS	Linear combination of body kinematic measures	Statistical: (1) define the fall indicator by a linear combination of two kinematic measures; (2) determine the weighting factor of the linear combination by optimization procedure; (3) The individual-specific control limit based on each fall indicator was used for fall detection	Slip induced falls	60 Young adults	SEN = 97.3 %, SPE = 99.2 %	Not specified
Kianoush et al.	[17]	Radio frequency	Difference of RF signal fluctuation between ADLs and falls	MLM: (1) RF power fluctuation was received by the wireless link in the cover area; (2) Hidden Markov Model (HMM) was trained by the RF signal perturbations; (3) The output from the HMM model, was used to distinguish falls from other activities	2 SMF: fall from chairs; fall from stand	2 Young adults	100 % Accuracy	Not specified
Lee et al.	[18]	A 9-axial IMU, attached to waist	Negative downward vertical velocity of trunk	Single THD based on the trunk velocity: THD = 1.2 m/s for fall vs. ADL, THD = 1.4 m/s for fall vs. Non-fall	5 Types of falls (involuntary and simulated): slip-induced fall; trip-induced fall; fall from hit or bump; misstep fall; sit-to-stand fall; falls due to fainting; stand-to-sit fall	11 Young male adults were asked to mimic the falling behavior of elderly	SEN = 97.4 %, SPE = 99.4 %, (falls vs. ADL), SEN = 95.2 % SPE = 97.6 % (falls vs. near-fall)	184–231 ms

*MCS* motion capture system, *THD* threshold, *ACCM* accelerometer, *GYRO* gyroscope, *SMF* simulated falls, *SEN* sensitivity, *SPE* specificity, *ADLs* activities of daily living

## Fall detection apparatus

The apparatus used in the existing pre-impact fall detection can be generally classified into the context aware systems and wearable sensors [15]. Five studies were based on the context aware technology. Among them, four studies used the motion capture system [12, 13, 26, 47], and one was based on the radio frequency signal analysis [17].

In the fall detection studies using the motion capture system, kinematic fall detection indicators were determined by reflective markers placed at anatomic landmarks of the human body. The trajectories of these reflective markers were tracked by motion capture cameras mounted in fixed locations. The biomechanical convention to calculate the body kinematic measures based on the motion capture system has been well established. Therefore, the major advantage of using the motion capture system for fall detection is that fall detection indicators can be accurately determined. However, it is practically impossible to implement the motion capture system in real life applications. Therefore, the motion capture system can be only used for fall detection model development and evaluation.

Kianoush et al. [17] introduced a novel context-aware technology to detect falls in pre-impact phase. Their technology was based on tracking the fluctuation of wireless radio-frequency (RF) signals. They found that fall events would cause abnormal perturbations on the RF signal which can be discerned by a hidden Markov model. The wireless RF signal was ubiquitous, and no attachable markers or wearable sensors were required. These features made this fall detection technology nonintrusive.

A limitation of the context-aware based fall detection is that it is restricted by space in the application. The motion capture system always has limited capture volume. Kianoush’s RF based technology also required wireless network deployment within the fall detection area. In addition, both the motion capture system and the RF based technology are expensive. Thus, related research and applications were still restricted to experimental settings. Lower cost context-aware technologies for fall detection have been introduced recently, such as the vision-based technology [19], camera image processing technology [27], acoustic based technology [20], and Microsoft Kinect [42]. However, to our knowledge, no one has used these technologies in pre-impact fall detection. This can definitely be a direction for future research.

The majority of existing pre-impact fall detection systems (17 out of 21) are wearable-sensor-based. Due to the advancement in microelectronics and wireless communication technology, wearable micro-electro-mechanical systems (MEMS), such as accelerometers and gyroscopes, become small, light-weight and low-cost [35]. They are capable of capturing body movement unobtrusively and allow kinematic measurements to be monitored over extended space and time. This makes them suitable for pre-impact fall detection.

A few studies implemented fall detection by using a single type of wearable sensors. For example, fall detection solutions from Bourke et al. [8]; Lindemann et al. [21]; Shan and Yuan [40] and Tong et al. [44] relied on accelerometers only, while in Nyan et al. [32] and Bourke and Lyons [9], gyroscopes were the only measuring device for fall detection. The use of a single type of sensors can significantly reduce complexity and computational demand of the fall detection system. However, it also has adverse effects on fall detection. For instance, it has been argued that acceleration signals alone might not be able to detect falls accurately due to that such signals cannot effectively and efficiently differentiate falls from fall-like activities (e.g., successful balance recovery from external perturbations, and jumping) [14, 34].

Compared to using a single type of wearable sensors, integrated inertial measurement units (IMUs), which typically consist of a tri-axial accelerometer and a tri-axial gyroscope, have become more popular in pre-impact fall detection applications. There might be three reasons behind this trend. First, the recent development in MEMS technology facilitated the development of low-cost, small-sized IMU chips with low energy consumption. This makes the implementation of wearable IMUs much easier than before. Second, the IMUs can provide more data than a single type of sensors and allow using multiple fall detection indicators, which could improve the fall detection accuracy. Third, integrated IMUs allow the sensor fusion algorithm, such as the Kalman Filter [49] or extended Kalman Filter [38], to be used to correct errors in both accelerometer and gyroscope data, and thus could result in more accurate estimation of fall detection indicators.

There is an emerging trend of using wearable sensors in fall detection research [15]. The wearable sensors have some advantages over the context-aware systems. First, the wearable sensors can be used to detect falls in extended space. Second, wearable sensors have much lower cost than context-aware systems. Third, wearable sensors are easier to implement than the context aware systems. In particular, wearable sensors often have wireless communication features which allow them to communicate easily with smart phones or other internet-enabled devices, and do not require additional infrastructure installation.

However, there are still some limitations in wearable-sensor-based fall detection. First, people under the fall detection surveillance are required to wear the sensors all the time. This might result in low user compliance because people sometimes might forget to wear them. Second, the data stability are lower than the context-ware system. This can be affected by many factors like insufficient battery power, and the alarm transmission might be affected by data lost in wireless communication. Third, some fall detection indicators cannot be measured directly by the wearable sensors, and have to be obtained on an estimation basis. For instance, body segment velocity variables have to be estimated by the integration of acceleration signals from accelerometers. There are inevitably some errors resulted from such estimation procedure.

In a recent study, Liu and Lockhart [23] proposed an integrative ambulatory measurement framework that combined the motion capture data and IMU data to detect falls in the pre-impact phase. This was the first attempt to incorporate both the context-aware based system and wearable sensors simultaneously in pre-impact fall detection. In this framework, the data from the motion capture system was used as a reference to facilitate the development of an individual-calibrated fall discriminant function. Data from wearable IMUs was used as the input of this function to search the optimal thresholds that can distinguish falls from activities of daily living (ADLs). The evaluation results showed that the fall detection performance was enhanced with such an integrative system [22].

## Fall detection indictors

Fall detection indicators refer to the variables selected to discriminate falls from non-fall activities in fall detection algorithms. Among the reviewed articles, all except Kianoush et al. [17] defined fall detection indicators by using human body kinematics. The kinematic measures used to define fall detection indicators are typically classified into two categories: segment translational measures and segment rotational measures. Among the translational measures, trunk velocity [7, 9, 12, 13]; Lee et al. [18, 47, 48] and trunk accelerations [1, 26, 40, 41, 43, 44, 49] were the most widely used fall detection indicators. Head acceleration [13, 21] and upper arm velocity [13] were also used to define fall detection indicators. Fall detection indicators defined by rotational measures included angular rate of the sternum [33], angular rate of the waist [32, 41, 43] and trunk [22, 23], and segment orientation of the trunk [22, 23] and thigh [31, 32]. Most existing studies used a single kinematic measure to define the fall detection indicator. Only a few monitored more than one kinematic measures in their fall detection applications [12, 32, 47]. In particular, Wu [47] and Nyan et al. [32] both implemented ‘AND’ logic to link the selected kinematic measures, where falls were considered to be detected only when all the selected kinematic measures were beyond predefined thresholds. Hu and Qu [12] used a linear combination of trunk vertical velocity and shank frontal velocity as the fall detection indicator. The linear combination was considered as the simplest format of combination that made the fall detection implementation easy. Their results showed that such combination improved fall detection performance as compared to that using a single kinematic measure.

The selection of fall detection indicators was closely related to where to place fall detection sensors. The most commonly chosen body site was the waist area. Seven different research groups attached the wearable sensors on either the anterior or posterior side of the waist area to monitor the lower trunk kinematics. The chest (or upper trunk) area was another popular choice. Four research groups attached the sensor to the chest. The upper and lower trunk became preferred body sites for fall detection sensors, most probably because these two body areas are close to the whole-body center of mass. Researchers also considered the head [21], underarm [33] and thigh [32] for fall detector placement.

Some fall detection indicators cannot be measured directly by the wearable sensors. Instead, they were estimated from the sensor output. For example, the accelerometers can only measure the acceleration with the effect of gravity, which needs to be calibrated with the sensor orientation information to obtain the free acceleration. Besides, body segment velocity and orientation can only be calculated by integration of acceleration data (from accelerometer) and angular rate data (from gyroscope), respectively. For such integration procedure, there is always the drift problem due to the error accumulation in the integral results. Many approaches have been proposed to solve the drift problem, such as Kalman filters [25], the advanced integration methodology [3], and the optimization approach [11]. However, these methods have only been tested for normal activities. To our knowledge, no studies have attempted to use the aforementioned methods to estimate body kinematics during acute activities like falls. Future studies need to be carried out in this direction.

## Fall detection algorithms

Threshold-based algorithms appeared to be the simplest algorithm utilized in pre-impact fall detection research. With the application of threshold-based algorithms, a fall is considered to be detected if the selected fall detection indicators are beyond a pre-defined threshold. Otherwise, the activity is classified as a non-fall activity. Threshold-based algorithms are computationally efficient which allows them to be easily implemented in real-time applications. However, setting an appropriate threshold was always difficult. Typically, a higher threshold would result in fewer false alarms but more misdetection of falls; whereas a lower threshold would lead to less misdetection but more false alarms. Almost all the current threshold-based techniques face this dilemma. Some researchers defined the threshold by using the maximum readings of fall detection indicators during non-fall activities [7]. However, the selected non-fall activities in the experimental settings cannot be inclusive of all possible non-fall activities, and thus the threshold determined in this way may not sufficiently address the problem.

Machine learning algorithms, including support vector machine [1], hidden Markov model [41]; hidden Markov model [17, 44] and artificial neural networks [38], were also used in pre-impact fall detection research. A training period was required in the machine learning algorithms. In the training period, the data collected during non-fall activities were usually used to facilitate the feature extraction and activities classification. Machine learning algorithms are more computationally intensive compared to the threshold-based algorithms, and thus might lead to longer detection time.

Recently, Hu and Qu [12, 13] have presented a novel fall detection algorithm that was based on the statistical process control chart. In this model, statistical process control chart that was specified by upper and lower control limits was used to monitor fall detection indicator time series. The control limits were individual-specific as they were based on the data collected from each individual. Similar as the threshold-based approach, a fall was considered to be detected if the monitored fall detection indicator went beyond the range defined by the upper and lower control limits. Otherwise, the activity was considered to be normal. The limitation of this study, however, was that only slip-induced falls were tested.

## Types of falls for fall detection evaluation

Most studies used simulated falls, where the participants were asked to fall voluntarily. Forward, backward and sideway falls were the most common simulated falls. Falls during sit-to-stand transition [31, 32], and falls during stair negotiation [40] were also simulated in the laboratory for fall detection research. Most of fall accidents in real life are unexpected and involuntary in nature. Movement patterns during simulated falls must be different from the realistic unexpected falls. Thus, using simulated falls may result in over-rated fall detection evaluation results. Bagalà et al. [2] reported that the fall detection sensitivity with real-life fall data were much lower than that using simulated fall data. This was partially due to that the threshold calibrated on simulated fall signals might not be suitable for real-life fall scenarios.

Instead of simulated falls, some researchers used involuntary falls for fall detection evaluation [1, 12, 13, 22, 23, 26]. For example, realistic slip-induced falls were used in Hu and Qu [12, 13]. However, this study was limited by only one type of fall. It is worth noting that Aziz [1]; Sabatini et al. [38]; Lee et al. [18] used both voluntary falls (e.g. sit-to-stand fall and fall from fainting) and involuntary falls (e.g. falls from slips, trips and hit/bump). Their results can be better generalized into real-life situations than the other studies. The direction of future work in terms of selecting proper types of falls for fall detection evaluation is to establish a database about the involuntary falls in real life for fall detection development and evaluation.

It is also important to note that fall data were typically collected from younger adults instead of older adults in fall detection research due to safety and ethics concern. However, older adults have different postural control characteristics when being exposed to external perturbations initiating falls [24], and may hit the ground sooner than younger adults during falls [47]. Therefore, the model evaluation results based on younger adults was in general overestimated.

## Fall detection performance

The performance of pre-impact fall detection is mainly assessed from two perspectives: accuracy and efficiency. The fall detection accuracy was typically measured by sensitivity and specificity. Sensitivity is determined by the ratio of the number of successfully detected falls over the total number of falls. Specificity is determined by the ratio of the number of successfully detected non-fall activities over the total number of non-fall activities.

The sensitivities reported in the reviewed studies ranged from 80 to100 %. The sensitivity (=80 %) in Sabatini et al. [38] was the lowest. The rest studies can achieve sensitivity above 90 %. A few studies reported 100 % sensitivities, including Lindemann et al. [21], Nyan et al. [33], Bourke et al. [8], Wu and Xue [48], Shi et al. [41], Shan and Yuan [40], Tong et al. [44] and Liu and Lockhart [22, 23]. The specificities reported in the reviewed articles were from 85.6–100 %. The lowest specificity was reported in Aziz et al. [1]. A few studies reported 100 % specificities, including Bourke et al. [7, 8]; Nyan et al. [31, 32]; Shi et al. [41]; Shan and Yuan [40] and Sabatini et al. [38].

Note that it is not reasonable to conclude the best fall detection models just based on the reported sensitivity and specificity, as the fall data for evaluation are quite different across studies. In particular, the number of participants and the number of falls and other activities in model evaluation varied in different studies. These factors did affect the reported accuracy. Some studies, like Lindemann et al. [21] and Kianoush [17] involved only one or two participants, and reported relatively high fall detection accuracy. Besides, as mentioned earlier, the types of fall selected in the model evaluation also affect the reported fall detection accuracy. For the sake of comparison among different fall detection models, it is important to unify the standard such as the number of falls and other activities involved and the selection of fall types in the model evaluation.

Lead time and/or detection time are typically used to assess the efficiency of fall detection. Lead time was defined by the time interval between when the fall was detected and fall impact, and accounts for the time for protective measures to be activated to protect the fall victims from fall impacts. Thus, the longer the lead time is, the better the fall detection performance is. The reported lead time were from 40 to 750 ms. Shi et al. [41] proposed airbag technology for preventing fall impacts. The inflation time of the airbag was reported to be approximately 130 ms. In order to be effective in avoiding a fall impact, the lead time of fall detection should be longer than 130 ms.

Detection time was the time difference between the fall initiation and fall detection. This parameter is also used to indicate how rapid the fall detection system responds to a fall. A better fall detection performance is associated with a smaller detection time. Liu and Lockhart [22, 23] and Martelli et al. [26] reported a mean detection time of 255 ms and 351 ms, respectively. Hu and Qu [13] reported a mean detection time range between 620 and 710 ms. As the time interval between heel-strike and fall impact can be estimated to be around 900 ms [5, 30], these fall detection models can provide sufficient time for triggering fall protection device.

## Conclusion and future work

Research on pre-impact fall detection has been developed rapidly in recent years. This paper aims to have a systematic review on this topic. We reviewed some key aspects in the existing pre-impact fall detection research, including the fall detection apparatus, fall detection indicators, fall detection algorithms and types of falls for fall detection evaluation. We also reported the performance of the existing fall detection models.

There are some limitations in the current pre-impact fall detection research. First, the pre-impact fall detection is still limited by current technology. The context-aware systems often have high computational demand. In addition, such systems are expensive, difficult to implement, and restricted by space. The wearable sensors are limited by their sensor noise, especially when the fall detection indicators need to be estimated from the raw sensor output. Some wearable sensors are still bulky and intrusive that might lead to low user compliance. Second, the selection of appropriate fall detection indicators is essential for achieving desirable fall detection performance; however, there is no much empirical and theoretical evidence for the most appropriate fall detection indicators. Third, the current pre-impact fall detection research is limited by their external validity. The falls used for evaluating the fall detection model are often simulated. It is difficult to generalize the experimental results obtained from a simulated fall to the fall accidents that actually happen in real life. Realistic involuntary falls should be used in fall detection model development and evaluation.

Future work should be carried out to address these limitations. First, a low-cost context-aware system with an extended capture volume and robust classification algorithm, or a small-sized wireless IMU system with advanced sensor fusion algorithm (which lead to less error in the sensor output) and low-energy consumption should be developed in the near future. Second, the optimal body site for sensor placement needs to be further investigated. Future work should be carried out to assess fall detection performance with different fall detection sensor placement schemes. Lastly, to ensure the external validity, it is imperative to setup a database for realistic falls and ADLs.
